# Synthesis optimization of ZrO_2_ nanostructures for photocatalytic applications

**DOI:** 10.55730/1300-0527.3551

**Published:** 2023-02-21

**Authors:** Filiz BORAN, Merve OKUTAN

**Affiliations:** Department of Chemical Engineering, Faculty of Engineering, Hitit University, Çorum, Turkey

**Keywords:** Zirconia, photocatalytic application, synthesis optimization, particle size, crystal phase

## Abstract

This study aims to optimize the synthesis of semiconductor zirconia (ZrO_2_) nanoparticles for future photocatalytic applications in degradation of pollutants in wastewater under ultraviolet (UV) light. The synthesis procedure of ZrO_2_ nanoparticles was optimized by examining the effects of synthesis methods (ultrasound-assisted, hydrothermal method in an autoclave and conventional precipitation), reaction time (2, 6, 10, and 18 h), ambient pH (3, 7, 10, 13), and surfactant type (anionic, cationic, and non-ionic), on the particle size and crystal phase of the nanomaterial. The characterization of the synthesized samples was performed by X-ray diffraction (XRD), Fourier transform infrared spectroscopy (FTIR), energy-dispersive X-ray spectroscopy (EDS), high-contrast transmission electron microscopy (HR-TEM), and transmission electron microscope (TEM) analysis. Consequently, to synthesize ZrO_2_ nanoparticles with the smallest particle size and monoclinic/tetragonal phase, the experimental conditions were optimized as ultrasound-assisted synthesis method, pH 10, and 6 h reaction time without surfactant. Moreover, percentage yield, particle size, crystallinity, monoclinic and tetragonal volumes of ZrO_2_ nanostructures synthesized under optimized conditions were determined as 39.40%, approximately 9 nm, 46.77, 79%, and 21%, respectively. It has been determined that the particle sizes can be kept under control by controlling the phase composition of ZrO_2_ nanostructures by optimizing the synthesis parameters. Structural and morphological characterization results can be correlated to the photocatalytic application, showing the potential of this material for the photodegradation of organic dye pollutants.

## 1. Introduction

Considering the requirement for drinking and utility water across the world, it is critical to remove the dye and various organic pollutants that originate from different industrial wastes from limited water resources. Among the methods that can be used for saving water and preventing environmental pollution, photodegradation, which has advantages, such as not creating secondary pollution, simplicity, providing fast results, and being environmentally friendly, compared to traditional methods, has become an attractive research area in recent years. The photocatalytic reaction is a process where a semiconductor photocatalysis absorbs sunlight to degrade pollutants in water and at least two photochemical reactions take place on the photocatalyst. It is well known that metal oxide nanomaterials, which have unique optical, electronic, and magnetic properties, are used effectively in this process [1–3].

Nanostructured zirconia (ZrO_2_), which is one of the most industrially important transition metal oxides, stands out owing to its excellent chemical and physical properties such as low thermal conductivity, thermal stability, high refractive index, electrical and optical properties, chemical inertness, nontoxicity, biocompatibility, high fracture toughness and polymorphic structure [4–7]. Although its wide energy band in the range of 5 to 5.8 eV limits the use in photocatalysis, its strong oxidation property due to the highly negative conductivity band provides the capacity of creating holes to this material and makes it a potential candidate in this application [8,9].

There are several methods to obtain ZrO_2_ nanomaterials, which has three polymorphic structures at atmospheric pressure as monoclinic, tetragonal, and cubic, such as sol-gel and hydrothermal methods [8,10–13]. Obtaining pure tetragonal and monoclinic nanocrystals with a size of 10 nm or less among these three polymorphs is quite challenging and depends on many synthesis parameters [14]. For instance, the preparation of ZrO_2_ with conventional precipitation from aqueous solutions of zirconyl salts often leads to a mixture of stable monoclinic (m-ZrO_2_) and metastable tetragonal (t-ZrO_2_) forms. Moreover, because of the large volume change (about 47%), the phase transformation between monoclinic and tetragonal polymorph hinders the unique properties of bare ZrO_2_, which will be used in direct application. Such destructive phase transition can be avoided by stabilizing t-ZrO_2_ with appropriate cationic doping. Therefore, suitable preparation procedures should be used to obtain the desired crystalline form of ZrO_2_ for specific applications [15].

The effect of polymorphic structure on photocatalytic activities of ZrO_2_ has been investigated and reported in several studies in the literature. Basahel et al. reported that the degradation rate of methyl orange was higher for m-ZrO_2_ (low surface area) than that of ZrO_2_, which had tetragonal and cubic phases (high surface area). Although it is known that a photocatalyst, which has a high surface area, increases dye adsorption and subsequent photocatalytic activity, it has been reported that the adsorption coefficient is related to the amount of dye adsorption on a catalyst. This can be explained by the fact that a low surface area material with a high adsorption coefficient can adsorb as much material per catalyst as a high surface area material with a low adsorption coefficient [16]. In another study, Teeparthi et al. reported that the white ZrO_2_ crystals, which contained a mixture of monoclinic and tetragonal phases, played a dominant role in determining the catalytic efficiency in methylene blue degradation [17]. According to the literature, the efficiency of the photocatalytic process is directly related to the charge carrier units and energy band gaps of nanomaterials, which are affected by the crystallinity and the size of the nanomaterial [1,2]. It is well known that if process conditions such as solution pH, concentration, reaction temperature, reaction time, and solvent type are carefully maintained, ZrO_2_ particles of desired shapes and sizes can be obtained [18]. In addition, surfactants could be used in size and shape controlled nanoparticle synthesis. Surface active agents are related to surface adsorption, which enables nanomaterials to have the desired shape and size. More specifically, it is associated with the adsorption of surfactant molecules on the planes of nucleating centers. It is possible to produce various nanostructures, including nanospheres, nanotubes, and nanorods, with cationic, anionic, nonionic, and zwitterionic-based surfactants that can contain polar and nonpolar groups together [19–21].

This study differs from its counterparts in the literature due to the controlling of the synthesis process via parameter optimization to obtain nanosize ZrO_2_ which has both mixed phases and a particle size of less than 10 nm in a narrow size distribution. Therefore, in the present study, the synthesis conditions of ZrO_2_ were investigated and optimized, as it had the smallest size and the best morphology for future photocatalytic applications. For this purpose, ZrO_2_ was synthesized starting from Zirconium dichloride oxide octahydrate (ZrOCl_2_.8H_2_O) by using three different methods as conventional precipitation, ultrasound assisted synthesis, and hydrothermal method in an autoclave. For the chosen method, the effects of reaction time, ambient pH, and surfactant as anionic, cationic, and non-ionic on particle size and polymorphic structure were investigated as synthesis parameters. X-ray diffraction (XRD), Fourier transform infrared spectroscopy (FTIR), Energy-dispersive X-ray spectroscopy (EDS), high-contrast transmission electron microscopy (HR-TEM), and transmission electron microscope (TEM) analyses were performed for the confirmation and characterization of the structure.

## 2. Materials and methods

### 2.1. Materials

ZrOCl_2_.8H_2_O with 98% purity was purchased from ABCR. Sodium hydroxide (NaOH) was obtained from Carlo Erba Reagenti. SDS (sodium dodecyl sulfate, 99.5%, anionic), TPAB (tetrapropylammonium bromide, 98%, cationic), and PEG (polyethylene glycol, nonionic, Mw: 8000 g/mol) as surfactants were obtained from Advanced Diagnostics & Research and Aldrich Chemistry, respectively. All chemicals were used without purification.

### 2.2. Methods

#### 2.2.1. Synthesis of ZrO_2_ nanostructures

0.1 M ZrOCl_2_.8H_2_O was dissolved in 50 mL of distilled water (Milli-Q Direct 8, 18.2 MΩcm). After mixing with a magnetic stirrer ( Heidoph, MR Hei-Tec (EU), Germany) for 30 min at 500 rpm to be a homogeneous solution, 5 M NaOH solution was dropped in the resulting solution to adjust the pH = 10 measured with a pH meter (Thermo Scientific, Orion Star A111). It was then mixed for 6 h at 70 °C in an ultrasonic bath (Sonica Ultrasonic Cleaner, Soltec, Sweep System, 50/60 Hz, 1000 W, Italy). Next, the samples were collected by centrifugation (J.P. Selecta, Centronic-BL II, Spain) at 10,000 rpm for 15 min and washed with distilled water until the pH of the supernatant turned to 7. Finally, the obtained samples were dried for 16 h at 80 °C in an oven (Ecocell) and calcined in a muffle furnace (MSE Furnace/ATM_1700_8). The muffle furnace was heated in the temperature range from room temperature to 600 °C at a heating rate of 5 °C min^−1^ and held at 600 °C for 2 h under nonatmosphere-controlled conditions (Schema). Subsequently, the samples were stored in glass vials at room temperature before the characterization. The above ultrasound-assisted procedure was repeated for different parameters, including synthesis methods (hydrothermal synthesis in an autoclave and conventional precipitation), reaction times of 2, 6, 10, and 18 h, ambient pH (3, 7, 10, 13), and surfactant type (PEG8000, TPAB, and SDS). Surfactants were added at 0.2 mM (5% molar weight of Zr ions) before pH adjustment.

#### 2.2.2. Characterization of synthesized samples

FTIR of the synthesized samples were recorded using KBr pellets via a Mattson 1000 model spectrometer in the wave number range of 400–4000 cm^−1^. The amounts of Zr and O in the structures were determined with EDS using a LEO 440 computer controlled digital model scanning electron microscope (SEM) device. The morphological properties of the synthesized samples were investigated using a JEOL brand JEM 2100F Model HR-TEM device in the Central Laboratory of Middle East Technical University and a JEOL JEM 1220 Model TEM in the Central Research Laboratory Application and Research Center of Eskişehir Osmangazi University. XRD analysis was made with a Bruker brand D8 advance model X-ray diffractometer using CuKα radiation (35 kW, 15 mA, 1.541871 Å) with a scanning speed of 2 °/min. The average particle size of the ZrO_2_ nanostructures was determined using HR-TEM micrographs with ImageJ 1.53e image analysis program and XRD analysis data with the Scherrer equation (Eq. 1). In the calculation of the average crystallite particle size of ZrO_2_ nanoparticles (d_XRD_), the full widths of the ZrO_2_ reflection planes at the peak (2θ) half-height and the factor 0.89 K were used.


Eq. 1 $$d_{XRD}=\frac{0.89\lambda }{\beta cos\theta },$$

where λ, β, and θ are the X-ray wavelength, the peak half-height full widths and the Bragg angle, respectively [22].

Using the XRD data, the monoclinic ratio (X_m_), monoclinic volume (V_m_), and tetragonal volume (V_t_) were also calculated from Eqs. 2, 3, and 4, respectively. The equation given for V_t_ is appropriate only for samples showing tetragonal and monoclinic polymorphs [10].


Eq. 2 $$X_{m}=\frac{\left[I_{m}(1\;1\;1)+I_{m}(1\;1\;\bar{1})\right]}{\left[I_{m}(1\;1\;1)+I_{m}(1\;1\;\bar{1})+I_{t}(1\;1\;1)\right]},$$

where I_m_ (1 1 1), I_m_ (1 1 1̄), and I_t_ (1 1 1) are peak intensities at 29°, 31°, and 30°, respectively.


Eq. 3 $$V_{m}=\frac{1.311X_{m}}{1+0.311X_{m}}\times 100,$$

Eq. 4 $$V_{t}=100-V_{m}.$$

To calculate the percentage yield (%), the synthesized samples were dried in a vacuum oven for 3 h at 100 °C to completely dry. The percentage yield was calculated based on the initial weight of raw material received and the weight of the final product after completely drying [10].

## 3. Results and discussion

### 3.1. The effect of synthesis method

#### 3.1.1. XRD and FTIR analysis results

**Figure 1:**
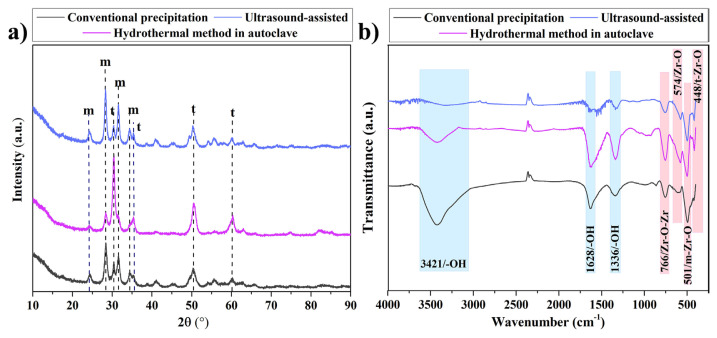
a) XRD diffractograms and b) FTIR spectra of ZrO_2_ nanostructures prepared with different synthesis methods.

XRD diffractograms of ZrO_2_ nanostructures synthesized using different synthesis methods are shown in Figure 1a. For all three methods, the diffraction peaks at 2θ = 24.1°, 28.2°, 31.5°, and 34.3° were assigned to the m-ZrO_2_ crystal phase [23,24], and weak peaks at 2θ = 30.2°, 35.2°, 50.6°, and 60.2° could be indexed to the pure t-ZrO_2_ crystal phase [16].

The percentage yield and the values obtained from the diffractograms such as particle size, crystallinity, V_m_, and V_t_ are given in Table 1. The ZrO_2_ nanostructures synthesized using the different synthesis methods showed percentage yields of 39.40%–41.77% varying in a narrow range. However, it was clearly seen that the particle size, V_m_, and V_t_ ratios of ZrO_2_ nanostructures were changed by using different synthesis methods. The particle size of the ZrO_2_ nanostructure synthesized using ultrasound-assisted method was smaller than the other samples. Moreover, the crystallinity of ZrO_2_ nanostructures ranged from 40.94% to 55.51%. It can be said that crystallinity decreases with decreasing peak intensities in XRD diffractograms (Figure 1a) [25]. As a result of calculations made from XRD diffractograms, it was observed that the crystallinity increased with the increase in the V_t_ ratio. According to these results, the type of synthesis method can affect the morphology of the synthesized sample.

FTIR spectra of ZrO_2_ nanostructures are shown in Figure 1b. The broad peaks seen at 3421 and 1628 cm^−1^ wavelengths belong to the −OH stretching and bonding vibrations of the water adsorbed on the nanostructure, respectively [4,18]. Similarly, the peak observed at 1336 cm^−1^ wavelength was thought to originate from the hydroxyl groups of hydrated molecules [26,27]. The peaks observed at a wavelength of 448 and 501 cm^−1^ were attributed to the tetragonal Zr-O band and the monoclinic Zr-O vibration, respectively [28–31]. The peaks at 766 and 574 cm^−1^ were related to Zr-O-Zr asymmetric stretching and Zr–O stretching, respectively [27,32].

#### 3.1.2. EDS analysis results

**Figure 2:**
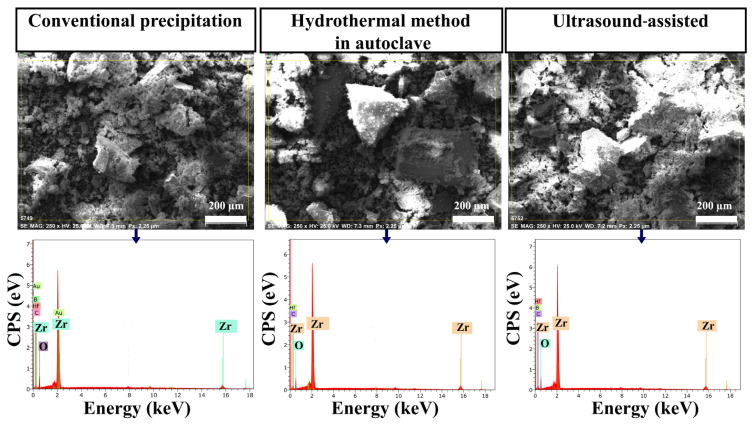
EDS images of ZrO_2_ nanostructures prepared with different synthesis methods.

The chemical composition of ZrO_2_ nanostructures was investigated using the EDS technique. According to these results, ZrO_2_ nanostructures prepared using different synthesis methods had very few impurities such as F, Hf, C, B, and Na (Figure 2). The C content of these samples probably came from the carbon band used for sample preparation, the device, and the ultrapure water used for synthesis. However, it is seen that ZrO_2_ samples were obtained in high purity in all three methods.

#### 3.1.3. HR-TEM analysis results

**Figure 3:**
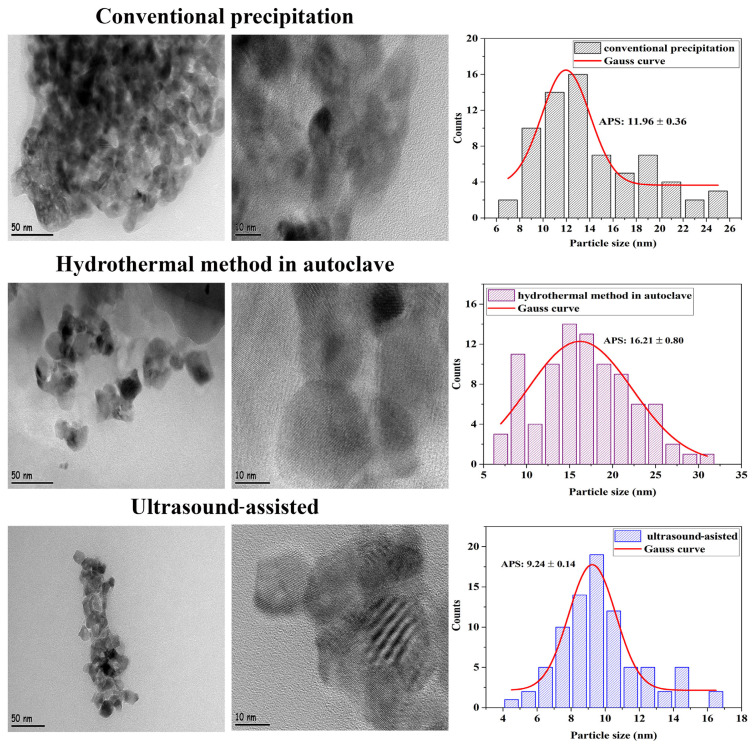
HR-TEM micrographs and particle size histograms of ZrO_2_ nanostructures prepared by different synthesis methods.

The particle distributions determined from the HR-TEM micrographs of ZrO_2_ nanostructures prepared with different synthesis methods are shown in Figure 3. It was seen that the samples synthesized with the hydrothermal method in the autoclave were intense in the range of 10–30 nm and had particles of different sizes. It was seen that the samples synthesized using the conventional precipitation method were intense in the range of 5–20 nm and had particles of different sizes. On the other hand, the particle sizes of the samples synthesized with the ultrasound-assisted method were most intense in the range of 5–15 nm and the smallest particle size was reached with this method. It was determined that the results obtained from HR-TEM confirmed the XRD results.

To examine the effect of the synthesis method type on the particle size and morphology of ZrO_2_ nanostructures, three methods were tried: conventional precipitation, hydrothermal method in an autoclave, and ultrasound-assisted. The smallest particle size and the best particle distribution were achieved with the ultrasound-assisted method. For this reason, studies on the effects of experimental conditions on the size and morphology of ZrO_2_ nanostructures were continued by choosing the “ultrasound-assisted” method as the synthesis method.

### 3.2. The effect of reaction time

#### 3.2.1. XRD analysis results

**Figure 4:**
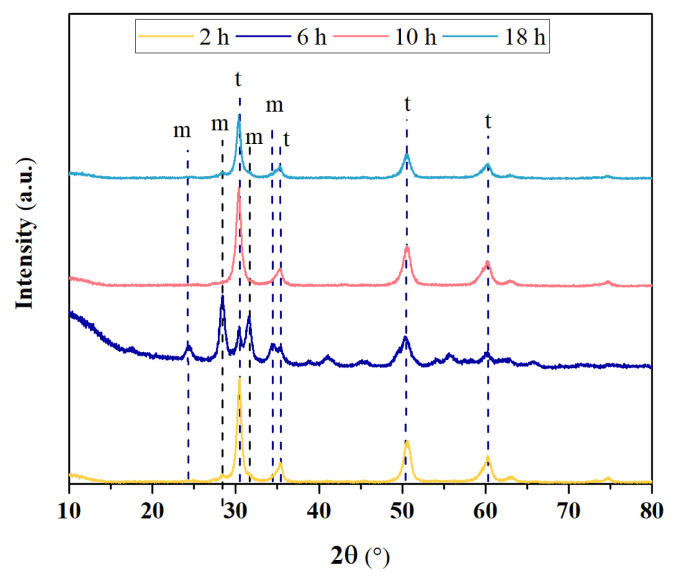
XRD diffractograms of ZrO_2_ nanostructures prepared with the ultrasound-assisted method with different reaction times.

In Figure 4, XRD diffractograms of ZrO_2_ nanostructures prepared with the ultrasound-assisted method at different reaction times are shown. For all example, the peaks at 2θ = 24.1°, 28.2°, 31.5°, and 34.3° corresponded to the m-ZrO_2_ crystalline phase [23,24]. Moreover, the weak peaks at 2θ = 30.2°, 35.2°, 50.6°, and 60.2° can be attributed to the pure t-ZrO_2_ crystal phase [16].

The percentage yield and the values obtained from diffractograms such as particle size, crystallinity, V_m_, and V_t_ are shown in Table 2. The ZrO_2_ nanostructures synthesized with the different reaction times showed percentage yields of 31.86%–39.40%, and the particle size changed in a narrow range of 9.21–11.91 nm. While synthesizing ZrO_2_ nanostructures with the ultrasound-assisted method, it was seen that the V_m_ and V_t_ ratios were affected by changing the time exposed to ultrasonic sound waves. It was observed that the crystallinity was affected by these changes in the V_t_ and the crystallinity of ZrO_2_ nanostructures ranged from 46.77% to 78.66%. In addition, the V_m_ ratio was raised by increasing the reaction time to 6 h, and the presence of only V_t_ occurred when the reaction time reached 10 h. According to this result, it can be said that the morphology and size of the synthesized sample can be controlled by changing the time exposed to ultrasonic sound waves while synthesizing with the ultrasound-assisted method.

#### 3.2.2. EDS analysis results

**Figure 5:**
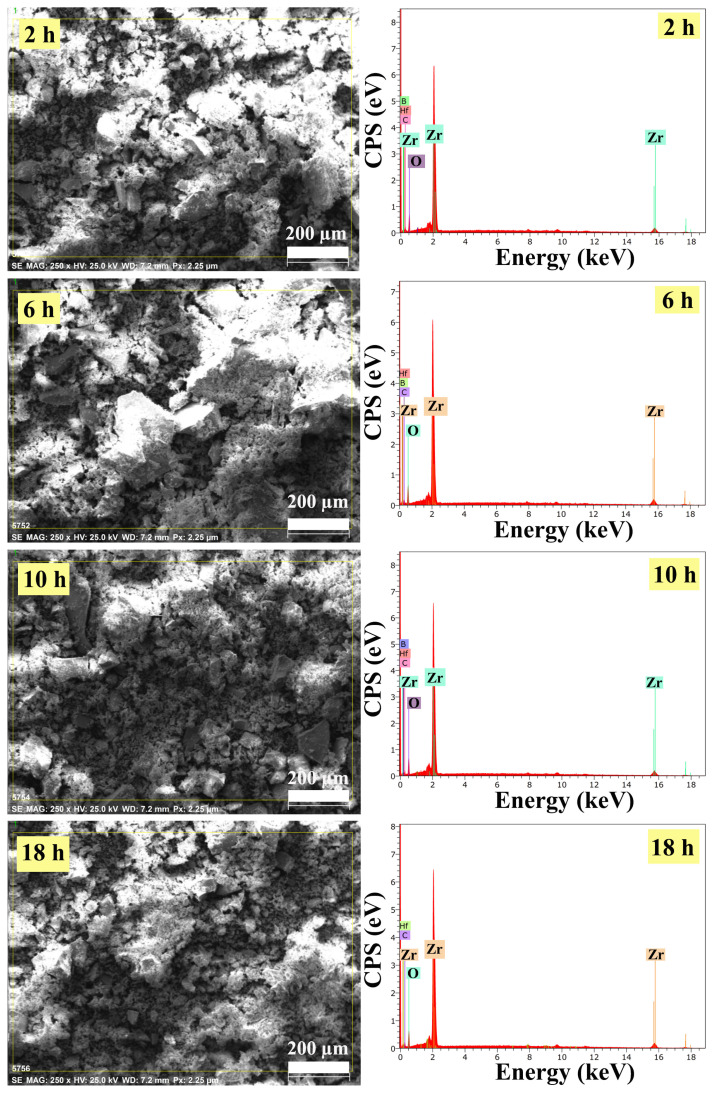
EDS images of ZrO_2_ nanostructures prepared with the ultrasound-assisted method with different reaction times.

EDS spectra were taken in the entire region belonging to the SEM images given in Figure 5. The spectrums of ZrO_2_ nanostructures prepared at different reaction times with the ultrasound-assisted method showed that the samples contained the elements of Zr and O. A few impurities were also found in EDS analyses, ascribable to synthesis residues and carbon band, corresponding to Hf, C, and B. The EDS results demonstrated that the main elements within all samples were Zr and O.

#### 3.2.3. HR-TEM analysis results

**Figure 6:**
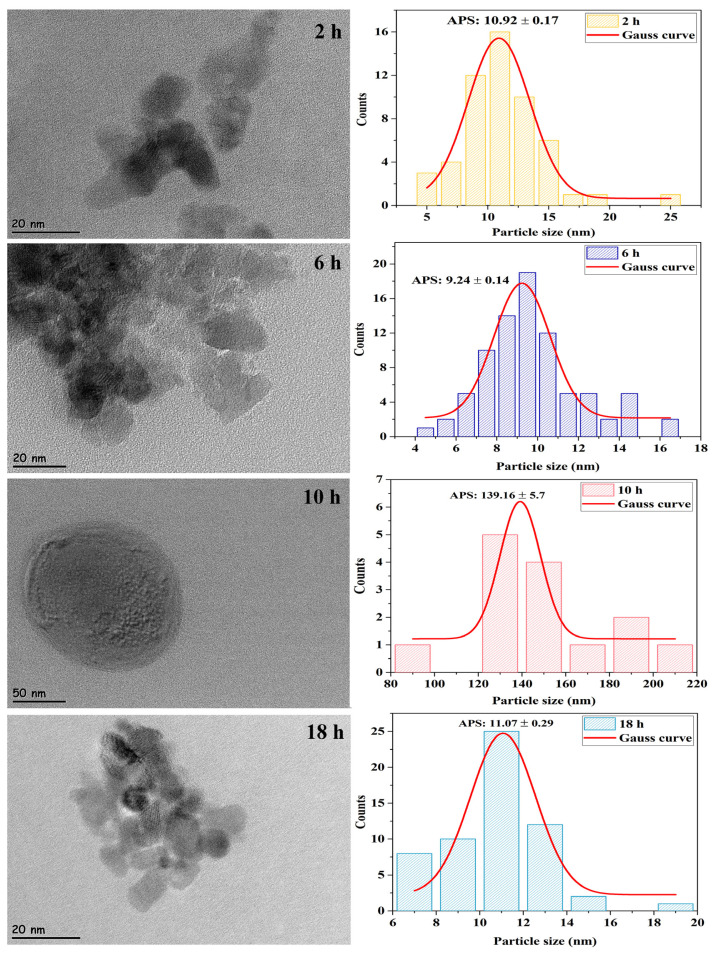
HR-TEM micrographs and particle size histograms of ZrO_2_ nanostructures prepared in different reaction times with the ultrasound-assisted method.

HR-TEM micrographs of ZrO_2_ nanostructures prepared at different reaction times with the ultrasound-assisted method are shown in Figure 6. It can be seen from the HR-TEM micrographs that the samples synthesized throughout 2 and 18 h were collected in a narrow area and agglomerated. Moreover, Figure 6 displays that the samples synthesized in 6 h reaction time were spread over a wider area and agglomeration can be partially avoided. The results obtained from HR-TEM micrographs for 2, 6, and 18 h reaction times were consistent with the XRD results. However, the sample synthesized in 10 h reaction time had a very large particle size (Figure 6) contrary to XRD analysis results.

Consequently, to examine the effect of reaction time on the particle size and morphology of ZrO_2_ nanostructures, four different reaction times as 2, 6, 10, and 18 h, were studied. According to all analysis results, it was concluded that the smallest particle size, the best particle distribution, and the morphology were reached in 6 h of reaction time. For this reason, studies on the effects of experimental conditions on the size and morphology of ZrO_2_ nanostructures were continued by choosing “6 h” as the reaction time.

### 3.3. The effect of ambient pH

#### 3.3.1. EDS analysis results

**Figure 7:**
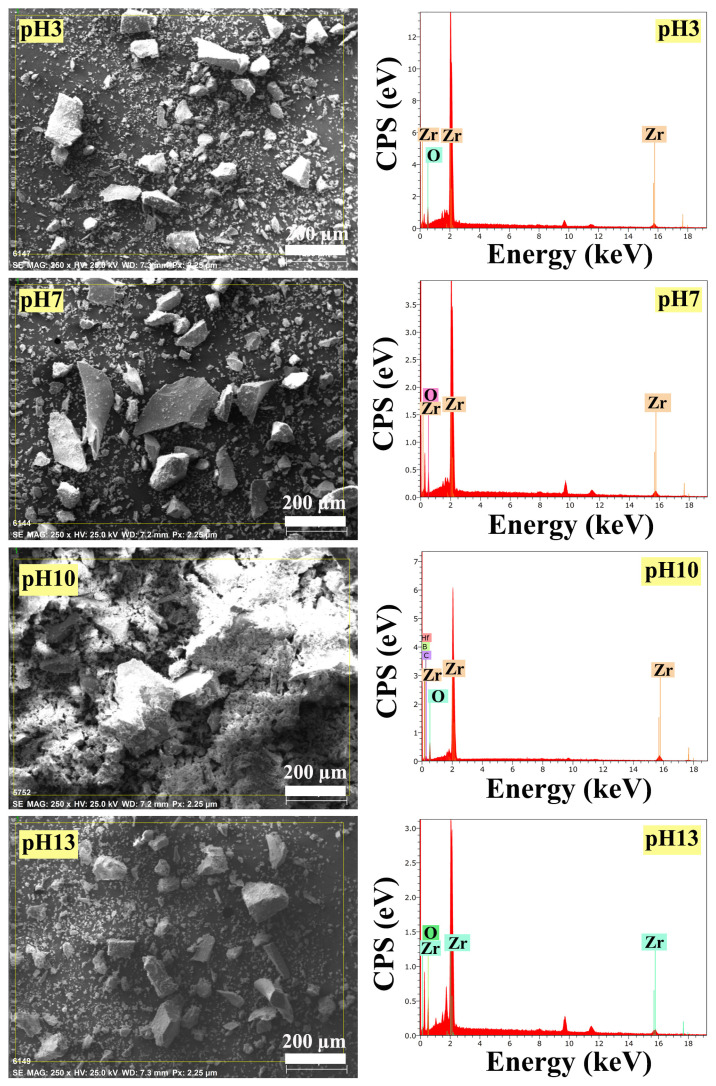
EDS images of ZrO_2_ nanostructures prepared with the ultrasound-assisted method with different ambient pHs for synthesis.

To determine the chemical composition of ZrO_2_ nanostructures, the EDS analysis was employed. As observed in Figure 7, the ZrO_2_ nanostructure obtained when the ambient pH was adjusted to 10 contained very few impurities such as Hf, C, and B. In addition, it was clearly identified that ZrO_2_ nanostructures prepared at other ambient pHs were composed of only Zr and O elements with higher purity.

#### 3.3.2. XRD analysis results

**Figure 8:**
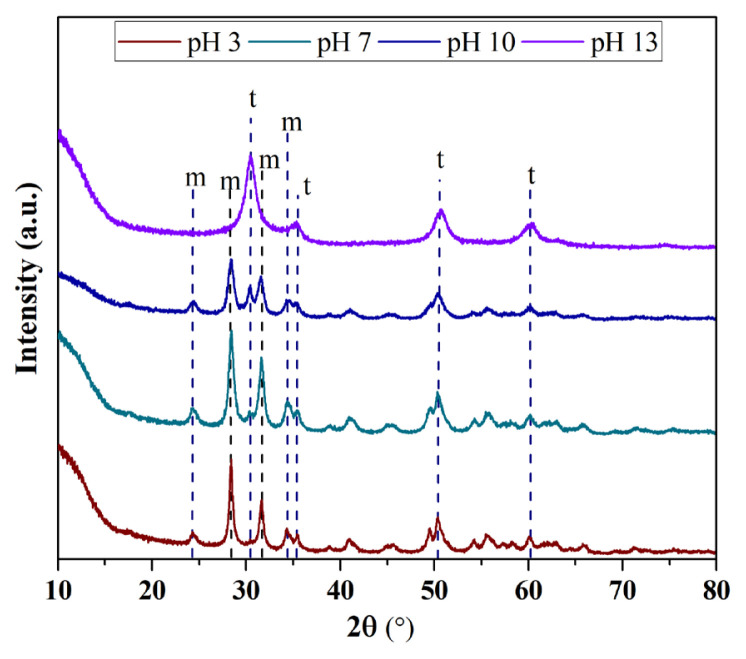
XRD diffractograms of ZrO_2_ nanostructures prepared with the ultrasound-assisted method with different ambient pHs for synthesis.

XRD diffractograms of ZrO_2_ nanostructures synthesized by changing the pH of the synthesis ambient are shown in Figure 8. For nanostructures prepared at ambient pH 3, 7, and 10, the peaks at 2θ = 24.1°, 28.2°, 31.5°, and 34.3° corresponded to the m-ZrO_2_ crystalline phase [23] and for all samples weak peaks at 2θ = 30.2°, 35.2°, 50.6°, and 60.2° can be attributed to the pure t-ZrO_2_ crystal phase [16]. As it is clearly understood from Figure 7, it was seen that only samples with tetragonal structure could be synthesized by keeping the ambient pH at 13. It was understood that ZrO_2_ nanostructures with both monoclinic and tetragonal structures can be synthesized by working at ambient pHs below this.

The values obtained from diffractograms such as particle size, crystallinity, V_m_, V_t_, and the percentage yield obtained after synthesis are displayed in Table 3. The ZrO_2_ nanostructures synthesized by using different ambient pHs for synthesis represented percentage yields of 4.23%–39.40% and the particle size varied in a range of 7.64–25.45 nm. Moreover, it was seen that the V_m_ and V_t_ ratios of ZrO_2_ nanostructures were influenced by varying the pH of the synthesis ambient. In addition, with the increase of the pH value to 13, it was observed that the V_t_ ratio was increased and the presence of only V_t_ occurred. The crystallinity of ZrO_2_ nanostructures ranged from 33.33% to 52.87%. Accordingly, it can be said that the morphology and size of the synthesized sample can be controlled by changing the pH of the synthesis ambient.

#### 3.3.3. TEM analysis results

**Figure 9:**
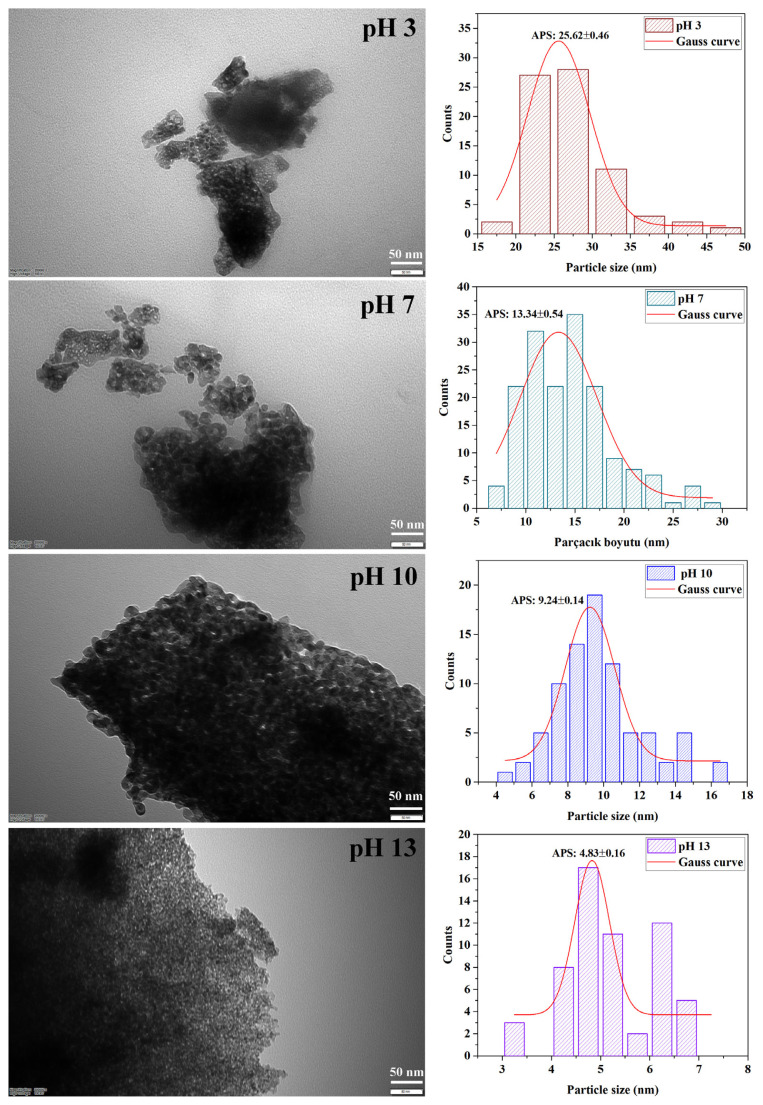
TEM micrographs and particle size histograms of ZrO_2_ nanostructures prepared in different ambient pHs for synthesis with the ultrasound-assisted method.

The morphology with the particle size distribution of the product prepared at different ambient pHs was further studied by TEM analysis in Figure 9. According to these images, spherical shaped samples at all pHs appeared to be tightly packed together. This can be attributed to the occurrence of agglomeration due to the very small size of the samples. When the XRD and TEM results were evaluated together, it was found that the monoclinic phase ratios of ZrO_2_ nanostructures decreased and the tetragonal phase ratios increased while the pH of the synthesis ambient was increased from 3 to 13. Notwithstanding, it was observed that the average particle sizes decreased. Average particle sizes obtained from HR-TEM images and XRD analysis results were consistent with each other.

In conclusion, it is clearly seen that the particle size can be reduced as the phase composition of the synthesized ZrO_2_ nanostructures turns into a single tetragonal phase. In some studies, it has been reported that ZrO_2_ nanostructures with a single tetragonal phase have smaller particle sizes [33]. Based on these results, it can be said that it is possible to control the particle size by controlling the phase composition of ZrO_2_ nanostructures by changing ambient pH for synthesis. Based on all these results, the smallest average particle size was reached when the ambient pH was above 10. However, while the particle morphology was both monoclinic and tetragonal up to pH 10, only tetragonal structure was formed at pH 13. Although the smallest particle size was reached at pH 13, only ZrO_2_ nanostructures with tetragonal structure were obtained. Therefore, it was decided to continue the study by selecting the synthesis ambient as pH 10, which allows the synthesis of ZrO_2_ nanostructures with small dimensions but containing tetragonal-monoclinic mixed phase.

### 3.4. Effect of surfactant type

#### 3.4.1. EDS analysis results

**Figure 10:**
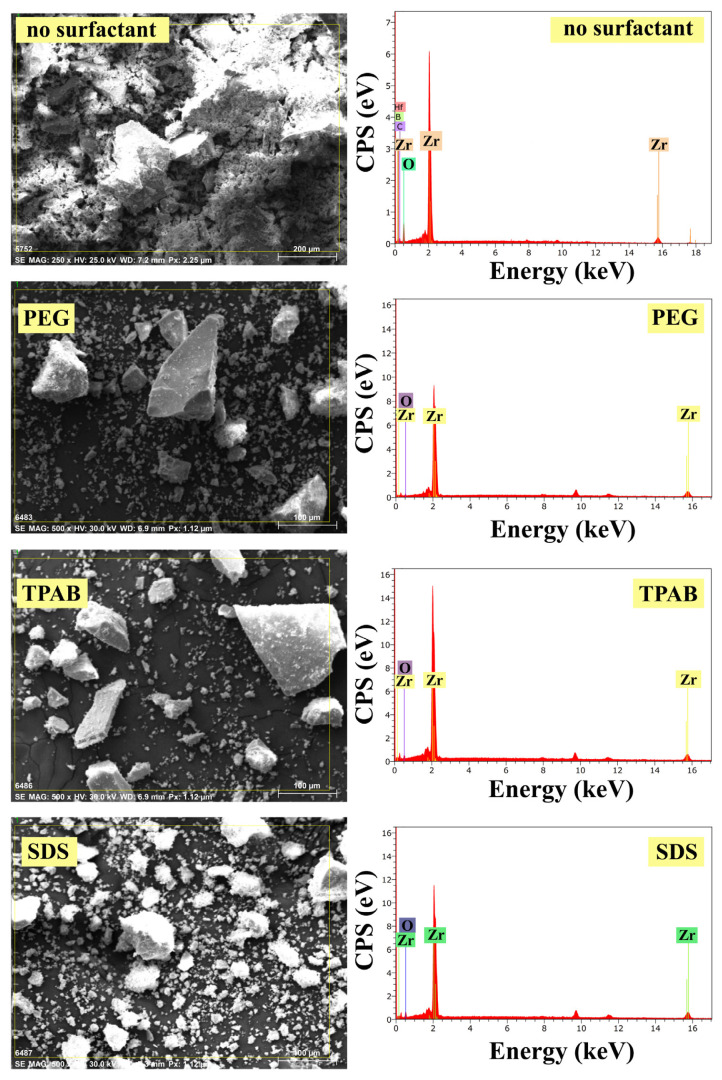
EDS images of ZrO_2_ nanostructures prepared with the ultrasound-assisted method with different types of surfactants.

The EDS analysis was used to determine the composition of ZrO_2_ nanostructures. As observed in Figure 10, the ZrO_2_ nanostructure prepared without the use of surfactant contained very few impurities such as Hf, C, and B. It was seen that ZrO_2_ nanostructures were prepared using different types of surfactants, composed of only Zr and O elements with higher purity.

#### 3.4.2. XRD analysis results

**Figure 11:**
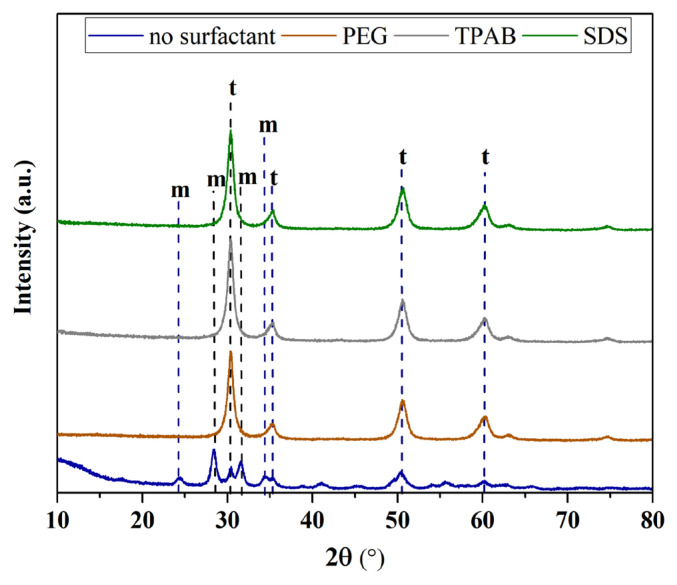
XRD diffractograms of ZrO_2_ nanostructures prepared with the ultrasound-assisted method with different types of surfactants.

Studies on the structural properties of ZrO_2_ nanostructures synthesized using different types of surfactants were done by XRD analysis. Figure 11 displays the XRD pattern of the ZrO_2_ nanoparticles. For nanostructures prepared without surfactant, the diffraction peaks were indexed to the monoclinic ZrO_2_ crystal phase with the characteristic peaks at 2θ = 24.1°, 28.2°, 31.5°, and 34.3° [23,24] and weak peaks at 2θ = 30.2°, 35, 2°, 50.6°, and 60.2° for all of the samples can be ascribed to the pure t-ZrO_2_ crystal phase [16]. As clearly seen in Figure 11, the samples with only tetragonal structure can be synthesized by adding any type of surfactant to the synthesis ambient. However, it is understood that ZrO_2_ nanostructures with both monoclinic and tetragonal structures can be synthesized when the surfactant is not used.

Moreover, the amount of formed crystalline phases and particle size were defined using XRD analysis and these results with the percentage yield obtained after synthesis are displayed in Table 4. The ZrO_2_ nanostructures synthesized by using different types of surfactants represented percentage yields of 39.04%–43.20%, and the particle size varied in a range of 8.02–9.16 nm. In addition, the crystallinity of ZrO_2_ nanostructures ranged from 46.77% to 71.67%. Notwithstanding, it was found that 79% and 21% of the obtained structure without surfactant was dedicated to monoclinic and tetragonal crystal structure, respectively. However, it was seen that only the presence of tetragonal crystalline phase was formed by adding PEG8000, TPAB, and SDS surfactants to the synthesis ambient of ZrO_2_ nanostructures. Therefore, the morphology and size of the synthesized sample can be controlled by adding any surfactant to the synthesis ambient.

#### 3.4.3. TEM analysis results

**Figure 12:**
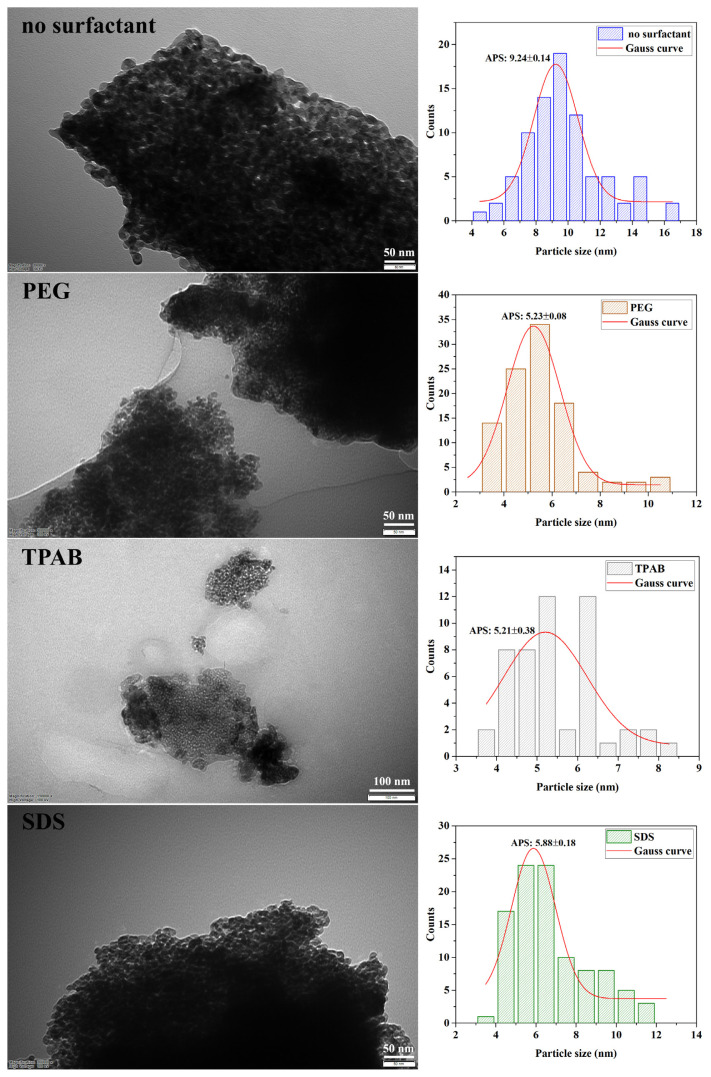
TEM micrographs and particle size histograms of ZrO_2_ nanostructures prepared using different types of surfactants with the ultrasound-assisted method.

The morphological structure and particle size distribution of ZrO_2_ nanostructures prepared by using three different types of surfactants, PEG8000, TPAB, and SDS, were determined by TEM analysis. As seen from the HR-TEM images in Figure 12, all samples had a spherical structure and were tightly packed together.

It was seen from the particle size distribution graphs obtained from the TEM micrographs that the particle size of the sample obtained without surfactant was intense in the range of 7–11 nm. Moreover, the particle sizes of the samples were intense in the range of 3–7 nm, 4–8 nm, and 4–6.5 nm when PEG8000, SDS, and TPAB were used, respectively. Very small particle sizes below 11 nm were obtained from TEM analysis which confirmed the XRD results. Moreover, the smallest average particle size was reached by using surfactants. However, while the particle morphology was both monoclinic and tetragonal without using a surfactant, only tetragonal structure was formed by using a surfactant. Therefore, it seems appropriate not to use the surfactant in the experimental system that allows the synthesis of small-sized but tetragonalmonoclinic mixed-phase ZrO_2_ nanostructures.

## 4. Conclusion

It appears that the white ZrO_2_ crystal structure, which contains a mixture of different crystal structures (monoclinic, tetragonal, cubic, etc.), plays a dominant role in determining the photocatalytic efficiency. In this study, synthesis optimization of ZrO_2_ nanoparticles with different crystal structures and smallest particle sizes has been made. For this purpose, the synthesis procedure was optimized by changing synthesis methods, reaction time, ambient pH, and surfactant type. The morphology, crystal structure, and particle sizes of the synthesized samples were analyzed with XRD, FTIR, EDS, HR-TEM, and TEM. According to the characterization results, the synthesis conditions were selected to obtain the smallest particle size and the ZrO_2_ nanostructure containing tetragonal-monoclinic mixed phase and the study was continued. Then, the synthesis optimization was completed. As a result, experimental conditions were optimized under conditions of surfactant-free, pH 10, 6 h reaction time, and ultrasound-assisted synthesis method. Under these conditions, the particle size and phase ratio of the ZrO_2_ nanostructure were determined as 9.24 nm and 79%/21% monoclinic/tetragonal phase, respectively. It has been determined that it is possible to control the particle sizes by controlling the phase composition of ZrO_2_ nanostructures by optimizing the synthesis parameters. It was concluded that the ZrO_2_ nanomaterials prepared in this study can be envisioned as promising materials in future photocatalytic applications. In addition, it was observed that ZrO_2_ nanoparticles with an average particle size of about 5 nm with only tetragonal phase were formed by adding any type of surfactant to the synthesis ambient under optimized conditions. For this reason, it is thought that this study with such ZrO_2_ nanomaterials will also benefit many researchers and application areas apart from photocatalytic applications.

## Figures and Tables

**Schema:**
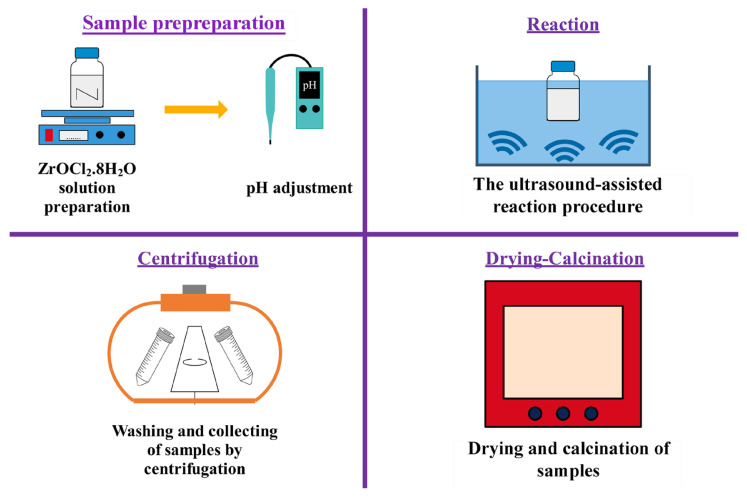
Synthesis step of ZrO_2_ nanoparticles.

**Table 1:** Percentage yield, particle size, crystallinity, V_m_, and V_t_ of ZrO_2_ nanostructures synthesized using different synthesis methods.

Synthesis methods	Percentage yield (%)	Particle size (nm)	Crystallinity (%)	V_m_ (%)	V_t_ (%)
Ultrasound-assisted	39.40	12.3	46.77	79	21
Conventional precipitation	41.77	13.1	55.51	45	55
Hydrothermal method in autoclave	40.39	17.2	40.94	85	15

**Table 2:** Percentage yield, particle size, crystallinity, V_m_, and V_t_ of ZrO_2_ nanostructures synthesized using different reaction times with the ultrasound-assisted method.

Reaction times (h)	Percentage yield (%)	Particle size (nm)	Crystallinity (%)	V_m_ (%)	V_t_ (%)
2	32.86	10.49	74.84	11	89
6	39.40	11.91	46.77	79	21
10	31.86	9.21	78.66	0	100
18	32.89	11.25	75.22	15	85

**Table 3:** Percentage yield, particle size, crystallinity, V_m_, and V_t_ of ZrO_2_ nanostructures synthesized using different ambient pHs for synthesis with the ultrasound-assisted method.

Ambient pH	Percentage yield (%)	Particle size (nm)	Crystallinity (%)	V_m_ (%)	V_t_ (%)
3	4.25	25.45	33.33	82	18
7	37.57	17.33	37.49	90	10
10	39.40	11.91	46.77	79	21
13	37.37	7.64	52.87	0	100

**Table 4:** Percentage yield, particle size, crystallinity, V_m_, and V_t_ of ZrO_2_ nanostructures synthesized using different types of surfactants with the ultrasound-assisted method.

Surfactants	Percentage yield (%)	Particle size (nm)	Crystallinity (%)	V_m_ (%)	V_t_ (%)
SDS	43.20	8.38	71.40	0	100
TPAB	38.54	8.36	70.63	0	100
PEG800	39.04	8.02	71.67	0	100
No surfactant	39.40	9.16	46.77	79	21
